# Allelic Variants of *ARMC5* in Patients With Adrenal Incidentalomas and in Patients With Cushing's Syndrome Associated With Bilateral Adrenal Nodules

**DOI:** 10.3389/fendo.2020.00036

**Published:** 2020-02-07

**Authors:** Beatriz Marinho de Paula Mariani, Mirian Yumie Nishi, Ingrid Quevedo Wanichi, Vania Balderrama Brondani, Amanda Meneses Ferreira Lacombe, Helaine Charchar, Maria Adelaide Albergaria Pereira, Victor Srougi, Fabio Yoshiaki Tanno, Filippo Ceccato, Daniela Regazzo, Mattia Barbot, Gianluca Occhi, Nora Maria Elvira Albiger, Marcelo Vieira-Corrêa, Claudio Elias Kater, Carla Scaroni, José Luis Chambô, Maria Claudia Nogueira Zerbini, Berenice B. Mendonca, Madson Q. Almeida, Maria Candida Barisson Villares Fragoso

**Affiliations:** ^1^Unidade de Suprarrenal, Disciplina de Endocrinologia e Metabologia, São Paulo, Brazil; ^2^Laboratorio de Hormônios e Genética Molecular LIM/42, Hospital das Clinicas da Faculdade de Medicina da Universidade de São Paulo, São Paulo, Brazil; ^3^Divisao de Urologia, Hospital das Clinicas da Faculdade de Medicina da Universidade de São Paulo, São Paulo, Brazil; ^4^Endocrinology Unit, Department of Medicine, Padova University/Hospital, Padova, Italy; ^5^Department of Biology, University of Padova, Padova, Italy; ^6^Endocrinology Division, Department of Medical and Surgical Sciences, University of Padua, Padua, Italy; ^7^Adrenal and Hypertension Unit, Division of Endocrinology and Metabolism, Department of Medicine, The Federal University of São Paulo Medical School, São Paulo, Brazil; ^8^Departmento de Patologia, Hospital das Clinicas da Faculdade de Medicina da Universidade de São Paulo, São Paulo, Brazil; ^9^Instituto Do Câncer Do Estado de São Paulo, São Paulo, Brazil

**Keywords:** adrenal nodules, *ARMC5*, allelic variants, Cushing's syndrome, adrenal incidentaloma

## Abstract

**Objective:** Germline *ARMC5* mutations are considered to be the main genetic cause of primary macronodular adrenal hyperplasia (PMAH). PMAH is associated with high variability of cortisol secretion caused from subclinical hypercortisolism to overt Cushing's syndrome (CS), in general due to bilateral adrenal nodules and rarely could also be due to non-synchronic unilateral adrenal nodules. The frequency of adrenal incidentalomas (AI) associated with PMAH is unknown. This study evaluated germline allelic variants of *ARMC5* in patients with bilateral and unilateral AI and in patients with overt CS associated with bilateral adrenal nodules.

**Methods:** We performed a retrospective multicenter study involving 123 patients with AI (64 bilateral; 59 unilateral). We also analyzed 20 patients with ACTH pituitary independent overt CS associated with bilateral adrenal nodules. All patients underwent germline genotyping analysis of *ARMC5*; abdominal CT and were classified as normal, possible or autonomous cortisol secretion, according to the low doses of dexamethasone suppression test.

**Results:** We identified only one pathogenic allelic variant among the patients with bilateral AI. We did not identify any pathogenic allelic variants of *ARMC5* in patients with unilateral AI. Thirteen out of 20 patients (65%) with overt CS and bilateral adrenal nodules were carriers of pathogenic germline *ARMC5* allelic variants, all previously described. The germline *ARMC5* mutation was observed in only one patient with bilateral AI; it was associated with autonomous cortisol secretion and showed to be a familial form.

**Conclusion:** The rarity of germline *ARMC5* mutations in AI points to other molecular mechanisms involved in this common adrenal disorder and should be investigated. In contrast, patients with overt Cushing's syndrome and bilateral adrenal nodules had the presence of *ARMC5* mutations that were with high prevalence and similar to the literature. Therefore, we recommend the genetic analysis of *ARMC5* for patients with established Cushing's syndrome and bilateral adrenal nodules rather than patients with unilateral AI.

## Introduction

Primary macronodular adrenal hyperplasia (PMAH) is considered a rare cause of Cushing's syndrome (CS) and is generally due to bilateral adrenal nodules (1, 2). The clinical presentation is associated with the variability of cortisol secretion and hormonal exposure time (3–5). Patients are frequently diagnosed with PMAH when they are being examined for the cause of endogenous ACTH pituitary independent CS due to bilateral adrenal nodules. Until recently, it was believed that the clinical overt form of CS occurred only around the 5th and 6th decades of life. However, the subclinical presentation seems to occur frequently and generally these patients are undiagnosed (6, 7).

Inactive *ARMC5* germline mutations were identified in 55% and in 27% of familial and apparently sporadic forms of PMAH, respectively (8–15). Nevertheless, an increasing number of familial cases have been reported after the identification of *ARMC5* mutations as causative of PMAH, and the real incidence and frequency of PMAH are unknown.

Over the past 9 years, we have observed that the majority of Brazilian family members with PMAH present with mild symptoms of hypercortisolism, despite the presence of bilateral adrenal nodules and germline *ARMC5* mutations (8). In addition, ~30% of the index cases' relatives from one of the largest families presented with possible autonomous cortisol secretion and only one of the adrenal glands was clearly affected in these patients (8).

Unilateral adrenal nodules are usually identified in the context of adrenal incidentaloma (AI), which has an incidence that ranges from 1.9 to 4%, depending on the modality of the radiological imaging exam and the age of the patients (16, 17). In contrast, incidental bilateral adrenal nodules are rare and represent ~15% of all AIs (18). It is unknown which of these patients belong to the heterogenous spectrum of PMAH. We thought that this should be investigated. For this investigation, we focused on analyzing the frequency of the *ARMC5* allelic variants in a multicenter cohort of patients with adrenal incidentalomas (bilateral and unilateral adrenal nodules) or with bilateral adrenal nodules associated with adrenal ACTH pituitary independent Cushing's syndrome.

## Materials and Methods

The Ethical Committees from all centers involved (University of São Paulo, Brazil, University of Padova—Italy and University Federal of São Paulo—Brazil) approved this study and informed consent was applied for all adult patients.

All patients underwent abdominal CT scans and hormonal evaluations: basal plasma ACTH and serum cortisol after dexamethasone suppression test (DST).

In addition, free urinary cortisol and midnight cortisol (serum/salivary) levels were measured in patients with Cushing's syndrome.

### Dexametasone Suppression Test

The patients with adrenal incidentaloma were classified according to the DST as follows:

A) Normal values of serum cortisol (F) suppression (*F* ≤ 1.8 μg/dL or 50 nmol/L) after DST.
B) Possible autonomous cortisol secretion (1.8 μg/dL < *F* ≤ 5.0 μg/dL) after DST.
C) Autonomous cortisol secretion (*F* > 5.0 μg/dL) after DST.

### Cohort Composition

From 123 patients with AI, 71 were from the University of São Paulo; 45 were from the University of Padova; 7 were from the UNIFESP.

### Clinical Presentation

We classified the clinical presentation of patients in subclinical hypercortisolism or in overt Cushing's syndrome. Patients with subclinical hypercortisolism were diagnosed when they presented laboratory results of abnormal cortisol levels (possible/autonomous serum cortisol secretion after DST), without the so-called *specific* clinical signs of Cushing's syndrome (mainly the lack of: catabolic features; without proximal myopathy, skin fragility, and facial plethora). The overt Cushing's syndrome was defined by the presence of *specific* physical signs of hypercortisolism associated with abnormal cortisol results (i.e., possible/autonomous cortisol secretion after DST and increased 24 h urinary cortisol and suppressed ACTH levels).

The 123 patients were enrolled in 3 groups as follows:

**Group 1:** Patients with *bilateral adrenal incidentalomas:*
  64 patients −42 females; median age of 51.45 years of old [ranging from 16 to 64 years of age] and 22 males; median age of 54.21 years of old [ranging from 37 to 77 years of age].
**Group 2:** Patients with *unilateral adrenal incidentalomas:*
  59 patients −45 females; median age of 54.33 years of old [ranging from 34 to 79 years of age] and 14 males; median age of 59 years of old [ranging from 38 to 50 years of age].
**Group 3:** Patients with *ACTH pituitary independent Cushing syndrome and bilateral adrenal nodules:*
  20 patients −18 females; median age of 51.5 years of old [ranging from 35 to 69 years of age] and 2 males; median age of 52 years of old [ranging from 51 to 53 years of age].

### Abdominal CT Evaluation

All patients had an abdominal CT scan and an expert radiologist, who was blinded to the patients' molecular status of *ARMC5* performed the report. The nodules with the largest diameters and the number of nodules were considered on the CT scans for each adrenal gland. PMAH was defined morphologically based on the presence of bilateral adrenal nodules that were larger than 1 cm on the CT scans.

### Pathological Diagnosis

An expert pathologist (# MCNZ) confirmed the diagnosis of PMAH with the histological reports from all patients who underwent surgery.

### Molecular Analysis

We collected blood samples from 123 patients for the germline genetic analysis of *ARMC5*; the DNA was sequenced by Sanger sequencing to screen for the allelic variants of *ARMC5*. We used the Mutation Taster, Poly-Phen, Sift, PANTHER, VarSome, and ABraOM sites for variant prediction analysis, genomAD for variants frequency analysis (classified according to the American College of Medical Genetics), and we used the ENST00000268314 *ARMC5* isoform and NM_001105247.1- OMIM as references.

## Results

### Group 1—Patients With Bilateral Adrenal Incidentalomas (AIs)

**Figure 1:**
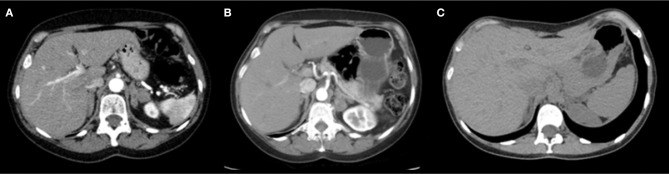
Abdominal CT scan with contrast showing. **(A)** Index-case: volumetric increase and diffuse nodular thickening of both adrenal glands, stable since 2011. **(B)** Index-case's sibling: bilateral enlargement of both adrenal glands with multiple hypoattenuating nodules, the largest measuring 2.5 cm (in the medial stem of the left adrenal), all with average attenuation of <10 HU in the non-contrast phase. Such nodules remain stable in relation to the tomographic study from 2015. **(C)** Index-case's niece: Both adrenal glands with preserved morphology and attenuation without identifiable focal lesions.

All 64 patients with bilateral AIs underwent DST with 1 mg of dexamethasone overnight. We classified 22/64 (34.4%) as having normal cortisol secretion; 27/64 (42.2%) as having a possible autonomous cortisol secretion and 15/64 (23.4%) as having autonomous cortisol secretion. Independent of the pattern of cortisol secretion, we identified the presence of non-pathogenic allelic variants of *ARMC5* in 84.4% (Annex Table 1). Only one patient had AI nodules with subclinical hypercortisolism and autonomous cortisol secretion presenting the germline pathogenic allelic variant c.1084C > T p.Arg362Trp in exon 3 of *ARMC5* (35^*^). During the familial segregation investigation, we identified that her sister and her niece were also carriers of the same germline mutation. The oldest of them presented bilateral adrenal nodules and possible cortisol secretion after DST (*F* = 4.4 μg / dL), the niece had a normal abdominal CT with normal suppression of cortisol after DST (*F* = 1.3 μg / dL) Table 1 and Figure 1.

**Table 1:** Hormonal data from the index-case and her relatives.

**Family**	**Age** **(yrs)**	**Basal serum cortisol** **(3.7–19.4 μg/dL)**	**Basal plasmatic ACTH** **(7.2–63.3 μg/dL)**	**Urinary cortisol** **(50–310 μg/24 h)**	**Midnight salivary cortisol** **(<0.12 μg/dL)**	**Cortisol after DST** **(<1.8 μg/dL)**
Index-case	61	16.5	8	20.7	0.11	**9.6**
Sister	59	12.5	10.4	20.6	0.10	**4.4**
Niece	26	14.9	8.5	N/A	0.40	< 1.3

*Bold indicates that the values are above upper limit*.

### Group 2—Patients With Unilateral Adrenal Incidentalomas

Among 59 patients with unilateral adrenal incidentalomas, all underwent DST evaluation. We classified 34/59 (57.6%) as having normal cortisol secretion; 18/59 (30.5%) as having a possible autonomous cortisol secretion and 7/59 (11.9%) as having autonomous cortisol secretion. Independent of the pattern of cortisol secretion, we identified the presence of non-pathogenic allelic variants of *ARMC5* in 69.5% of these patients (Annex Table 2).

**Figure 2:**
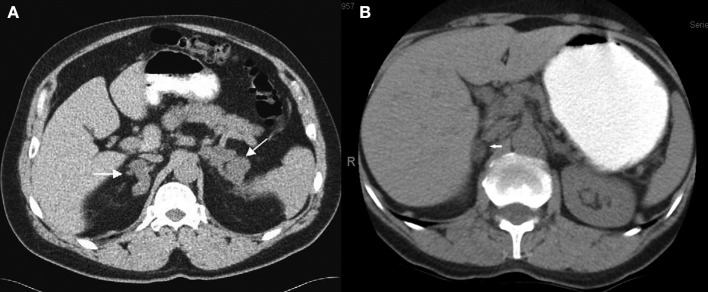
Abdominal CT scan with contrast showing the heterogenous presentation of the disease. **(A)** Bilateral adrenal nodules case #14. **(B)** Unilateral adrenal nodule—right gland case #20.

Considering the localization of the adrenal nodules, 38/59 (64.5 %) were located on the right side of the adrenal gland. Eight cases (13.5%) progressed to bilateral adrenal nodules in a time period of 10 months to 3 years Figure 2. Only one patient, who presented a contralateral adrenal nodule after 22 months of follow-up, showed an increase in serum cortisol levels post DST from 8.9 to 12.8 μg/dL. He maintained the midnight salivary cortisol levels and the 24 h urine free cortisol levels within the normal limits. Five of these patients who underwent adrenalectomy had the histological diagnosis confirmed as PMAH by the expert pathologist (# MCNZ).

### Group 3—Patients With Cushing's Syndrome ACTH Pituitary Independent With Bilateral Adrenal Nodules (Brazilian Cohort)

Patients were referred to our service (Tertiary Center—Hospital das Clinicas of FMUSP) to establish the differential diagnosis of endogenous Cushing's syndrome. All 20 patients presented with specific signs of Cushing's syndrome along with possible/autonomous cortisol secretion and low/suppressed plasmatic ACTH levels. Radiological imaging revealed bilateral adrenal involvement. All patients underwent surgical procedures to control the hypercortisolism. Only one patient underwent total bilateral adrenalectomy in the 80's. All the others underwent adrenal sparing surgery compounded by total unilateral adrenalectomy of the largest adrenal plus the partial contralateral adrenalectomy (adrenal sparing surgery). Patients developed secondary adrenal insufficiency and were required to receive hydrocortisone replacement until the full establishment of the hypothalamic-pituitary-adrenal axis. All patients were considered cured of hypercortisolism when the cortisol secretion levels were below 1.8 ug/dL after DST and without the necessity of cortisol replacement using cortisol levels over 19 ug/dL after 250 mcg iv of cosytropin test.

Thirteen out of 20 (65%) patients had germline pathogenic *ARMC5* allelic variants. From these index cases, we identified six new unrelated families, with 11 screened members, who were all carriers of germline *ARMC5* mutations (Annex Tables 3, Annex Tables 4). All relative members were classified as having possible autonomous cortisol secretion, according to cortisol levels after DST (Table 2).

**Table 2:** Hormonal data from the 143 patients.

**Characteristics**	**Bilateral** **AI**	**Unilateral** **AI**	**CS ACTH-pituitary independent**
Females (n)	42	45	18
Males (n)	22	14	2
DST (1 mg) F < 1.8 μg/dL	34.4%	57.6%	0%
DST (1 mg) 1.8 ≤ *F* ≤ 5 μg/dL *PACS*	42.2%	30.5%	15%
DST (1 mg) *F* > 5 μg/dL *ACS*	23.4%	11.9%	85%
Suppressed plasmatic ACTH Ref. (7.2–63.3 μg/dL)	42.2%	18.6%	90%
Elevated 24 h urinary free cortisol Ref. (50–310 μg/24 h)	12.2%	5.3%	37.5%
Elevated midnight salivary cortisol Ref. (< 0.12 μg/dL)	23.8%	32%	81.2%

*F, female; M, male; DST, dexamethasone suppression test V.O overnight*.

**Figure 3:**
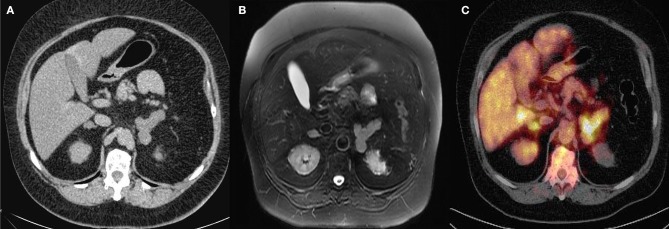
**(A)** Pre-contrast abdominal computed tomography (CT) scan (case # 3). **(B)** Post-contrast abdominal CT **(C)** Fluorine-18-fluorodeoxyglucose positron emission tomography 18F-FDG-PET/CT (case # 3) with Cushing's syndrome ACTH pituitary independent with bilateral adrenal nodules.

Six of the 11 patients underwent tomography exams and an abdominal ultrasound (3-month-old child; Figure 3); 4 of them had bilateral adrenal nodules, and 3 of them were normal. The four patients with bilateral nodules were examined with the DST and 3 of them presented autonomous cortisol secretion and 1 presented possible autonomic secretion.

**Figure 4:**
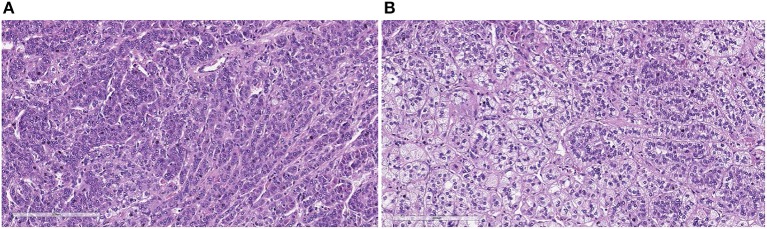
The histological sections show variable aspects, with areas with predominance of clear cells, vacuolated, with alveolar pattern **(A)** and others with predominance of compact cells with trabecular pattern **(B)** (H&E, X200).

Two out of 11 patients had Cushing's syndrome manifestation and underwent adrenal sparing surgery (Figure 4).

Seven patients with germline *ARMC5* mutations had no relatives screened, so they were considered apparently sporadic patients. They also presented autonomous cortisol secretion and six of them underwent surgery, due to Cushing's syndrome.

## Discussion

In this study we investigated the involvement of the *ARMC5* in three groups of patients, to look for pathogenic allelic variants: (1) patients with bilateral adrenal incidentalomas (with possible/autonomous cortisol secretion); (2) patients with unilateral adrenal incidentaloma (with possible/autonomous cortisol secretion); and (3) patients with overt Cushing's syndrome ACTH pituitary independent associated with bilateral adrenal nodules.

Several features suggest PMAH is predominantly an inherited disease and several familial forms have been recognized (7–15). Bilateral adrenocortical hyperplasia suggests a genetic mutation underlying pathogenesis in early on in embryogenesis affecting both adrenal glands although, non-synchronic presentation could also be observed. It is not clear if apparently sporadic cases of PMAH diagnosed as unilateral or bilateral adrenal incidentalomas, where familial clinical screening has not been performed, should be screened to *ARMC5* analysis. In addition, few studies that actively screened apparently unaffected relatives of patients with PMAH have shown affected individuals (8).

To the best of our knowledge, only one study has addressed the analysis of the *ARMC5* gene on bilateral adrenal incidentalomas, however, this was done in a small cohort (19). The authors did not identify any pathogenic allelic variants of *ARMC5*, even in cases with subclinical hypercortisolism, by DST (51% of cases) (19). Another study identified that ~25% of a cohort of 98 patients with sporadic and familial bilateral adrenal nodules, were carriers of *ARMC5* germline mutations. However, in this study, it is unclear whether the patients had adrenal incidentalomas or whether they were being examined due to over Cushing's syndrome (12).

In our study, among 123 patients with adrenal incidentaloma, 55% had abnormal serum cortisol levels after DST (Table 2). The majority of these patients had possible cortisol secretion. ~25% of patients with bilateral adrenal incidentalomas had autonomous cortisol secretion, compared to 12% of patients with unilateral adrenal incidentalomas. However, we are not sure whether those patients had PMAH or adenomas because they did not undergo surgical procedures.

In a single patient with a diagnosis of celiac disease who underwent abdominal CT imaging and was diagnosed with bilateral adrenal nodules, we identified the ARMC5 pathogenic allelic variant c.1084C > T p.Arg362Trp, which is located on exon 3 of ARMC5 (35^*^). This specific pathogenic allelic variant has already been described in patients with overt Cushing's syndrome (15, 20); in addition, this patient also had the c.2114C > T polymorphism, p.Ala705Val (rs11150624).

During familial segregation analysis, we observed that the patient's sister and her niece were also carriers of the same pathogenic variant, and both presented no signs of hypercortisolism. The suppression test results of her sister and her niece were compatible with possible autonomous cortisol secretion and normal cortisol secretion, respectively (cortisol levels of 4.4 and < 1.3 μg/dL, respectively).

The fact that the siblings did not develop any signs or symptoms of hypercortisolism by the time of the follow-up exam (5 years) is intriguing, although they were old enough (60 and 61 years) to present clinical features of hypercortisolism or at least metabolic syndrome. We noticed that the sister of the index case presented an acoustic meningioma, showing the link of PMAH and central nervous system meningiomas.

In addition to the fact that we identified the germline allelic variant of ARMC5 in a single patient with bilateral adrenal incidentaloma, we were also able to identify a new family that will now be in clinical follow-up related to the possibility of increasing hypercortisolism and the evaluation of the presence of meningiomas.

We performed a new *in silico* analysis (Annex Table 4), and some of the variants previously considered pathogenic or related to the disease changed to VUS. This change can occur once the analysis is made using different parameters. This classification continues to change, as the more we know about the variants, the more we understand whether they could be the reason for the pathogenicity.

The frequency of the variant in the population databases is also an issue to be considered, as PMAH is a heterogeneous disease and subclinical cases could be considered non-affected if not completely evaluated, making the definition of affected cases very complicated.

In the cohort of patients with bilateral and unilateral adrenal incidentalomas, we identified a higher frequency of non-pathogenic variants (~85 and 70%, respectively) of ARMC5 than that found in the cohort of Emms et al. (8%) (19). However, the meaning of this different frequency still needs to be investigated.

In contrast, pathogenic germline allelic variants of ARMC5 were identified in 65% of patients with ACTH pituitary-independent Cushing's syndrome with bilateral adrenal nodules, and 6 new families were diagnosed, improving their clinical care to avoid future health adversities.

The sensitivity of Sanger sequencing brings a limitation to our study as, to precisely detect gene alteration, the best methodology would be SNP array or target capture sequencing by NGS. Nevertheless, these methodologies are more expensive in clinical practice.

In conclusion, according to our results, the absence of pathogenic allelic variants in ARMC5 from patients with unilateral adrenal incidentalomas points out that other mechanisms may justify this common finding in clinical practice. However, we cannot imply that the cases without ARMC5 germline mutations could be adenoma or PMAH not related to ARMC5. The presence of the pathogenic allelic variant in a single patient with bilateral adrenal incidentaloma demonstrates the rarity of this form of clinical presentation.

According to our study, ARMC5 was shown to be a gene with high numbers of described non-pathogenic variants. The rarity of germline ARMC5 pathogenic allelic variants in AI leads to the hypothesis that other molecular mechanisms are involved in this common adrenal disorder and should be investigated. In contrast, the frequency of patients with overt Cushing's syndrome and bilateral adrenal nodules due to the presence of ARMC5 mutations was similar to that found in the literature. Therefore, we recommend the genetic analysis of ARMC5 for patients with established Cushing's syndrome and bilateral adrenal nodules rather than for patients with unilateral adrenal incidentaloma.

## Data Availability Statement

All datasets generated for this study are included in the article/Supplementary Material.

## Ethics Statement

The studies involving human participants were reviewed and approved by Comissão de Ética para Análise de Projetos de Pesquisa—CAPPesq Ethics Scientific Committee. Written informed consent was obtained from all patients.

## Author Contributions

BMM was the main author and provided all the molecular research and analysis. MN, IW, VB, AL, and HC contributed to the article providing the data (molecular and clinical) for some of the patients analyzed. MP, MA, and BBM also provided clinical data and contributed with their expertise for the study. VS, JC, and FT were the surgeons that made possible for us to collect the tumor tissue under right conditions. They also collaborated with their expertise to the study. FC, NA, DR, MB, CS, and GO contributed with DNA samples from Italy and clinical data. MV-C and CK contributed with DNA samples and clinical data from UNIFESP. MZ was the pathologist responsible for analyzing the tumor tissues and also contributed with her expertise to the elaboration of the project. MF is the leader and chief of the adrenal unit and helped with everything and at every part of the article.

### Conflict of Interest

The authors declare that the research was conducted in the absence of any commercial or financial relationships that could be construed as a potential conflict of interest.
