# Supplementary material for: Allelic Variants of ARMC5 in Patients With Adrenal Incidentalomas and in Patients With Cushing's Syndrome Associated With Bilateral Adrenal Nodules
Source: Front Endocrinol (Lausanne). 2020 Feb 7;11:36. doi: 10.3389/fendo.2020.00036 (PMC7019100; doi:10.3389/fendo.2020.00036)
Supplement: Supplementary file 1 [file Table_1.DOCX]

**Annex table 1.** Germline allelic variants from 64 patients with bilateral adrenal incidentaloma.

| Case Number # | Germline Allelic Variants | Varsome (Genome Interpreter) |
| --- | --- | --- |
| 1 | c.41T>A, p.Phe14Tyr, rs151069962, c.2058G>A, p.Ala686=, rs11863886 | rs151069962 (benign) BA1, BP4, BP6/ rs11150624 (benign): BA1, BP4 |
| 2 | c.2114C>T, p.Ala705Val, rs11150624 (hetero) | rs11150624 (benign): BA1, BP4 |
| 3 |  |  |
| 4 | c.41T>A; p.Phe14Tyr, rs151069962; c.1864+250C>T, rs11150624 | rs151069962 (benign) BA1, BP4, BP6/ rs11150624 (benign): BA1, BP4 |
| 5 | c.508A>G, p.Ile170Val, rs35923277 | rs35923277 (benign): BA1, BP4, BP6 |
| 6 | c.-306T>C, rs3813002; c.1864+250C>T, rs11150624 | rs3813002 (benign): BA1, BP4/ rs11150624 (benign): BA1, BP4 |
| 7 | c.41T>A, p.Phe14Tyr, rs151069962; c.1864+250C>T, rs11150624 | rs151069962(benign) BA1; BP4; BP6/ BP6, rs11150624 (benign): BA1, BP4 |
| 8 | c.2114C>T, p.Ala705Val, rs11150624 (hetero) | rs11150624 (benign): BA1, BP4 |
| 9 | ENST00000408912, c.106-152C>G, rs117600469; c.1520C>T; p.Pro507Leu; rs142376949 | rs117600469 (likely benign): BS1, BP4/ rs142376949 (likely benign): BS1, BP4 |
| 10 | c.41T>A, p.Phe14Tyr, rs151069962; c.1864+250C>T, rs11150624 | rs151069962 (benign) BA1, BP4, BP6/ rs11150624 (benign): BA1, BP4 |
| 11 | c.-753_-754 insC, c.438G>A, p.Arg146=, rs201280100 | rs201280100: BS1, BP7 |
| 12 | c.2114C>T, p.Ala705Val, rs11150624 (hetero) | rs11150624 (benign): BA1, BP4 |
| 13 |  |  |
| 14 | c.2058G>A, p.Ala686=, rs11863886 | rs771845578 (VUS): PM2, PP3 |
| 15 | c.2114C>T, p.Ala705Val, rs11150624 (hetero) | rs11150624 (benign): BA1, BP4 |
| 16 | ENST00000408912, c.106-152C>G, rs117600469; c.1520C>T; p.Pro507Leu; rs142376949 | rs117600469 (likely benign): BS1, BP4/ rs142376949 (likely benign): BS1, BP4 |
| 17 | c.583+26G>T, rs9921490 | rs9921490 (benign): BA1, BP4 |
| 18 | c.2114C>T, p.Ala705Val, rs11150624 (hetero) | rs11150624 (benign): BA1, BP4 |
| 19 | c.476-41G>A, rs762042093; c.1864+250C>T, rs11150624 | rs762042093 (VUS): BP4/ rs11150624 (benign): BA1, BP4 |
| 20 | c.407T>C, p.Leu136Pro, rs771845578, c.2058G>A, p.Ala686=, rs11863886 | rs11863886 (benign): BA1, BP7/ rs771845578 (VUS): PM2, PP3 |
| 21 | c.41T>A; p.Phe14Tyr, rs151069962; c.41T>A; | rs151069962(benign) BA1; BP4; BP6 |
| 22 | c.508A>G, p.Ile170Val; rs35923277 | rs35923277 (benign): BA1, BP4, BP6 |
| 23 | ENST00000408912, c.105+111G>A, rs114519904; ENST00000408912, c.125C>G, p.Thr42Arg, rs28451331; c.2058G>A, p.Ala686=, rs11863886 | rs114519904 (benign): BA1, BP4/ rs28451331 (benign): BA1, BP4/ rs11863886 (benign): BA1, BP7 |
| Case Number # | **Germline Allelic Variants** | **Varsome (Genome Interpreter)** |
| 24 | c.2114C>T, p.Ala705Val, rs11150624 (hetero) | rs11150624 (benign): BA1, BP4 |
| 25 | c.2114C>T, p.Ala705Val, rs11150624 (hetero) | rs11150624 (benign): BA1, BP4 |
| 26 |  |  |
| 27 | c.-306T>C, rs3813002; c.1520C>T; p.Pro507Leu; rs142376949; c.-306T>C, rs3813002; | rs142376949 (likely benign): BS1, BP4/ rs3813002 (benign): BA1, BP4 |
| 28 | c.2114C>T, p.Ala705Val, rs11150624 (hetero) | rs11150624 (benign): BA1, BP4 |
| 29 | c.41T>A, p.Phe14Tyr, rs151069962 | rs151069962(benign) BA1; BP4; BP6 |
| 30 | c.1864+250C>T, rs11150624 | rs11150624 (benign): BA1, BP4 |
| 31 | c.105+131C>T, rs113055278; c.41T>A, p.Phe14Tyr, rs151069962; c.2192C>G, p.Pro731Arg | rs113055278 (VUS): BP4/ rs151069962(benign) BA1; BP4; BP6 |
| 32 | c.1842C>G, p.Leu614=, rs55800131 | rs55800131 (benign): BA1,BP7/ |
| 33 | c.2114C>T, p.Ala705Val, rs11150624 (hetero) | rs11150624 (benign): BA1, BP4 |
| 34 | rs114519904, c.-913G>A; rs28451331, c.-652C>G; c.583+26G>T, rs9921490; c.1864+250C>T, rs11150624 | rs35923277 (benign): BA1, BP4, BP6/rs9921490 (benign): BA1, BP4, rs11150624 (benign): BA1, BP4 |
| 35* | c.1084C>T, p.Arg362Trp rs1385397608  c.2114C>T, p.Ala705Val, rs11150624 (hetero) | VUS PM2, PP3, PP1  rs11150624 (benign): BA1, BP4 |
| 36 | c.968G>C, p.Gly323Ala, rs35461188 | rs35461188 (benign): BA!, BP4 |
| 37 |  |  |
| 38 | rs9926717 A/G | rs9926717 (benign): BA1, BP4 |
| 39 | rs3813002 C/T | rs3813002 (benign): BA1, BP4 |
| 40 | rs151069962 T/A, F14Y | rs151069962(benign) BA1; BP4; BP6 |
| 41 |  |  |
| 42 | rs3813002 C/T | rs3813002 (benign): BA1, BP4 |
| 43 | rs3813002 C/T | rs3813002 (benign): BA1, BP4 |
| 44 | rs55800131 C/G | rs55800131 (benign): BA1,BP7/ |
| 45 | rs3813002 C/T | rs3813002 (benign): BA1, BP4 |
| 46 | rs3813002 C/T | rs3813002 (benign): BA1, BP4 |
| 47 | rs556386398 (c.2449C>T) | rs55800131 (benign): BA1,BP7/ |
| 48 | rs151069962 T/A, F14Y | rs151069962(benign) BA1; BP4; BP6 |
| 49 | rs3813002 C/T | rs3813002 (benign): BA1, BP4 |
| 50 | rs55800131 C/G | rs55800131 (benign): BA1,BP7/ |
| 51 | rs9926717 A/G | rs9926717 (benign): BA1, BP4 |
| 52 |  |  |
| 53 | rs3813002 C/T | rs3813002 (benign): BA1, BP4 |
| 54 | rs3813002 C/T | rs3813002 (benign): BA1, BP4 |
| 55 |  |  |
| 56 |  |  |
| 57 |  |  |
| 58 | rs3813002 C/T | rs3813002 (benign): BA1, BP4 |
| 59 | rs3813002 C/T | rs3813002 (benign): BA1, BP4 |
| 60 | rs3813002 C/T | rs3813002 (benign): BA1, BP4 |
| 61 | rs3813002 C/T | rs3813002 (benign): BA1, BP4 |
| 62 |  |  |
| 63 | rs9926717 A/G | rs9926717 (benign): BA1, BP4 |
| 64 | rs3813002 C/T | rs3813002 (benign): BA1, BP4 |
