# Supplementary material for: Allelic Variants of ARMC5 in Patients With Adrenal Incidentalomas and in Patients With Cushing's Syndrome Associated With Bilateral Adrenal Nodules
Source: Front Endocrinol (Lausanne). 2020 Feb 7;11:36. doi: 10.3389/fendo.2020.00036 (PMC7019100; doi:10.3389/fendo.2020.00036)
Supplement: Supplementary file 2 [file Table_2.DOCX]

**Annex table 2.** Germline allelic variants from 59 patients with unilateral adrenal incidentaloma.

| Case Number # | Germline Allelic Variants | Varsome (Genome Interpreter) |
| --- | --- | --- |
| 1 | c.2790G>A, p.Gly930=, | rs61734240 (VUS): BP7 |
| 2 |  |  |
| 3 | c.2114C>T, p.Ala705Val, rs11150624 (hetero) | rs11150624 (benign): BA1, BP4 |
| 4 | c.2790G>A, p.Gly930=, (hetero), rs61734240 | rs61734240 (VUS): BP7 |
| 5 | c.41T>A; p.Phe14Tyr; rs151069962; | rs151069962 (benign) BA1, BP4, BP6 |
| 6 | c.2114C>T, p.Ala705Val, rs11150624 (hetero) | rs11150624 (benign): BA1, BP4 |
| 7 | c.1641G>A, p.Ala547=, rs11150624 | rs11150624 (benign): BA1, BP4 |
| 8 | c.2073G>A; p.Pro691= , rs146763279 | rs146763279 (likely benign): BS1, BP7 |
| 9 |  |  |
| 10 | c.2114C>T, p.Ala705Val, rs11150624 (hetero) | rs11150624 (benign): BA1, BP4 |
| 11 | c.438G>A, p.Arg146=, rs201280100 | rs201280100: BS1, BP7 |
| 12 |  |  |
| 13 | c.1864+250C>T, rs11150624 | rs11150624 (benign): BA1, BP4 |
| 14 |  |  |
| 15 |  |  |
| 16 | c.513G>A, p.Gln171=, rs201474630 | rs201474630 (VUS): BP7 |
| 17 | Rs151069962 p.F14Y | rs151069962 (benign) BA1, BP4, BP6 |
| 18 | c.1842C>G, p.Leu709=, rs55800131; c.1864+250C>T, rs11150624 | rs55800131 (benign): BA1,BP7/ rs11150624 (benign): BA1, BP4 |
| 19 | rs35923277 p.I170V | rs35923277 (benign): BA1, BP4, BP6 |
| 20 | c.-306T>C, rs3813002 ; c.1864+250C>T, rs11150624 | rs3813002 (benign): BA1, BP4/ rs11150624 (benign): BA1, BP4 |
| 21 | c.1864+250C>T, rs11150624 | rs11150624 (benign): BA1, BP4 |
| 22 |  |  |
| 23 |  |  |
| 24 |  |  |
| 25 | c.1864+250C>T, rs11150624 | rs11150624 (benign): BA1, BP4 |
| 26 |  |  |
| 27 |  |  |
| 28 | rs28451331 SNP – 5’UTR - p.T42K | rs28451331 (benign): BA1, BP4 |
| 29 | c.1864+250C>T, rs11150624 | rs11150624 (benign): BA1, BP4 |
| 30 |  |  |
| 31 | c.1864+250C>T, rs11150624 | rs11150624 (benign): BA1, BP4 |
| 32 | c.174C>T, p.Ile58=,rs181081811; c.1842C>G, p.Leu614=, rs55800131, | rs181081811 (likely benign): PP3, BS1, BP7/ rs55800131 (benign): BA1, BP7 |
| 33 | ENST00000408912, c.105+111G>A, rs114519904 ; c.583+26G>T, , rs9921490 | rs114519904 (benign): BA1, BP4/ rs9921490 (benign): BA1, BP4 |
| 34 | c.1842C>G, p.Leu614=, rs55800131 | rs55800131 (benign): BA1, BP7 |
| 35 | c.1864+250C>T, rs11150624 | rs11150624 (benign): BA1, BP4 |
| 36 |  |  |
| 37 |  |  |
| 38 |  |  |
| 39 | c.1864+250C>T, rs11150624 | rs11150624 (benign): BA1, BP4 |
| 40 | c.1842C>G, p.Leu614=, rs55800131; (CTC>CTG), c.1864+250C>T, rs11150624 | rs55800131 (benign): BA1, BP7/ rs11150624 (benign): BA1, BP4 |
| 41 |  |  |
| 42 |  |  |
| Case Number # | **Germline Allelic Variants** | **Varsome (Genome Interpreter)** |
| 43 | rs3813002 C/T | rs3813002 (benign): BA1, BP4 |
| 44 | rs3813002 C/T | rs3813002 (benign): BA1, BP4 |
| 45 | rs3813002 C/T | rs3813002 (benign): BA1, BP4 |
| 46 |  |  |
| 47 | rs9926717 A/G | rs9926717 (benign): BA1, BP4 |
| 48 | rs377719718 GGCCT/GGCCT (homo) | rs377719718 (benign): BA1, BP4 |
| 49 | rs3813002 C/T | rs3813002 (benign): BA1, BP4 |
| 50 |  |  |
| 51 | rs3813002 C/T | rs3813002 (benign): BA1, BP4 |
| 52 | rs3813002 C/T | rs3813002 (benign): BA1, BP4 |
| 53 | rs3813002 C/T | rs3813002 (benign): BA1, BP4 |
| 54 | rs3813002 C/T | rs3813002 (benign): BA1, BP4 |
| 55 | rs9926717 A/G | rs9926717 (benign): BA1, BP4 |
| 56 | rs3813002 C/T | rs3813002 (benign): BA1, BP4 |
| 57 | rs3813002 C/T | rs3813002 (benign): BA1, BP4 |
| 58 | rs3813002 C/T | rs3813002 (benign): BA1, BP4 |
| 59 | rs3813002 C/T | rs3813002 (benign): BA1, BP4 |
