# Supplementary material for: Allelic Variants of ARMC5 in Patients With Adrenal Incidentalomas and in Patients With Cushing's Syndrome Associated With Bilateral Adrenal Nodules
Source: Front Endocrinol (Lausanne). 2020 Feb 7;11:36. doi: 10.3389/fendo.2020.00036 (PMC7019100; doi:10.3389/fendo.2020.00036)
Supplement: Supplementary file 3 [file Table_3.DOCX]

**Annex table 3.** Germline allelic variants from 20 patients with Cushing's syndrome ACTH pituitary independent with bilateral adrenal nodules.

| Case Number # | Germline Allelic Variants | Varsome  (Genome Interpretor) | Germline Allelic Variants | Varsome (Genome Interpretor) | Intervar  (Genome Interpretor) | Somatic Allelic Variants | Varsome (Genome Interpretor) |
| --- | --- | --- | --- | --- | --- | --- | --- |
| 1 |  |  | c.968G>A, p. Gly323Asp | VUS: PM2, PP3. | VUS: PM1, PM2 |  |  |
| 2 | c.583+26G>T, rs9921490; c.729C>T,p.Ala243=, rs114871627; c.1864+250C>T, rs11150624; c.2040T>C, p.Gly680=, rs116201073 | rs9921490 (**benign**): BA1, BP4/rs114871627 (likely benign): BS1, BP7/ rs11150624 (benign): BA1, BP4/ rs116201073 (benign): BA1, BP7 | c.281delC, p. Ser94Cysfs*43 | VUS: PM2 |  | No LOH |  |
| 3 | c.-173C>T, rs139912838; c.583+26G>T, rs9921490; c.1864+250C>T, rs11150624; c.2040T>C, p.Gly680=, rs116201073 | rs139912838 (likely benign): BS1, BP4/ rs9921490 (benign): BA1, BP4/ rs11150624 (benign): BA1, BP4/ rs116201073 (benign): BA1, BP7 | c.1960C>T; p.Arg654* | VUS: PM2, PP3. | Pathogenic: PVS1, PM2, PP3 | LOH |  |
| 4 | c.2114C>T, p.Ala705Val, rs11150624 (hetero) | rs11150624 (**benign**): BA1, BP4 | c.2692C>T, p.Arg898Trp, rs587777659 | VUS: PM2, PP3, PP5 | VUS: PM2, PP5 | c.766delT, p.Leu256* (hetero) Right adrenal and LOH left adrenal | VUS: PM2 |
| 5 | c.41T>A, p.Phe14Tyr, rs151069962 | rs151069962 (**benign**) BA1, BP4, BP6 | c.172dupA, I58Nfs*45 | VUS:PM2 |  |  |  |
| 6 |  |  | c.1094T>C, p.Leu365Pro, rs587777663 | VUS: PM2, PP3 | VUS: PM1, PM2, PP5 |  |  |
| 7 |  |  |  |  |  |  |  |
| 8 | c.-306T>C;T= 0,274/596; rs3813002 (het); + 12C>T (?) | rs3813002 (**benign**): BA1, BP4 |  |  |  |  |  |
| 9 | c.1864+250C>T, rs11150624 (homo) | rs11150624 (**benign**): BA1, BP4 |  |  |  | nl |  |
| 10 |  |  |  |  |  |  |  |
| Case Number # | **Germline Allelic Variants** | **Varsome**  **(Genome Interpretor)** | **Germline Allelic Variants** | **Varsome (Genome Interpretor)** | **Intervar**  **(Genome Interpretor)** | **Somatic Allelic Variants** | **Varsome (Genome Interpretor)** |
| 11 | c.1520C>T; p.Pro507Leu; rs142376949; (hetero)(CCG>CTG) T:0,009/19 | rs142376949 (**likely benign**): BS1, BP4 |  |  |  | nl |  |
| 12 | c.-306T>C;T= 0,274/596; rs3813002; (het); c.41T>A; (het) p.Phe14Tyr; rs151069962; A=0,029/63 | rs3813002 (**benign**): BA1, BP4/ rs151069962 (benign) BA1, BP4, BP6 |  |  |  |  |  |
| 13 | c.41T>A, p.Phe14Tyr, rs151069962, (A:0,043;T:0,957)(hetero) | rs151069962 (**benign**) BA1, BP4, BP6 |  |  |  |  |  |
| 14 | c.2114C>T, p.Ala705Val, rs11150624 (hetero) | rs11150624 (**benign**): BA1, BP4 | c.799C>T, p.Arg267*, rs369721476 | VUS: PM2, PP3 | Pathogenic: PVS1, PP3, PP5 | No LOH |  |
| 15 | c.508A>G; p.Ile170Val, rs35923277 | rs35923277 (**benign**): BA1, BP4, BP6 | c.1985C>A, p.Pro662Hist | VUS: PM2, PP3 | VUS: PM1, PM2 | c.809delC, Arg271Glyfs*7 (hetero) | VUS: PM2 |
| 16 | c.2114C>T, p.Ala705Val, rs11150624 (hetero) | rs11150624 (**benign**): BA1, BP4 | c.1090C>T, p.Arg364* | VUS: PM2, PP3 | Pathogenic: PVS1, PM2 e PP3 |  |  |
| 17 | c.2114C>T, p.Ala705Val, rs11150624 (hetero) | rs11150624 (**benign**): BA1, BP4 | c.391-392dupTT, p.Leu131Phefs*7 | VUS: PM2 |  |  |  |
| 18 | c.2114C>T, p.Ala705Val, rs11150624 (hetero) | rs11150624 (benign): BA1, BP4 | c.1094T>C, p.Leu365Pro, rs587777663 | VUS: PM2, PP3 |  |  |  |
| 19 | c.41T>A, p.Phe14Tyr, rs151069962; c.1864+250C>T, rs11150624 | rs151069962 (**benign**) BA1, BP4, BP6/ rs11150624 (**benign**): BA1, BP4 | c.172dupA, p.Ile58Asnfs*45 | VUS:PM2 |  |  |  |
| 20 | c.-1103G>A; rs115527944 | rs115527944 (**likely benign**): BS1, BP4 | c.2696dupG, p.Leu900Serfs*12 | VUS:PM2 |  | c.1325delA; p.Glu442Glyfs*19 (hetero) A (AD) e * c.1095_1102delGCGGGATGinsC, p.Arg366Leufs*7 (hetero) (AE) | VUS: PM2, PP3/ *VUS: PM2 |
