# Supplementary material for: Allelic Variants of ARMC5 in Patients With Adrenal Incidentalomas and in Patients With Cushing's Syndrome Associated With Bilateral Adrenal Nodules
Source: Front Endocrinol (Lausanne). 2020 Feb 7;11:36. doi: 10.3389/fendo.2020.00036 (PMC7019100; doi:10.3389/fendo.2020.00036)
Supplement: Supplementary file 4 [file Table_4.DOCX]

**Annex table 4.** *In silico* analysis of germline and somatic allelic variants from 20 patients with Cushing's syndrome ACTH pituitary independent with bilateral adrenal nodules.

| Allelic Variants | | | | | | | | |
| --- | --- | --- | --- | --- | --- | --- | --- | --- |
|  | **c.968G>A, p. Gly323Asp** | **c.281delC, p. Ser94Cysfs*43** | **c.1960C>T; p.Arg654***  **(Ref. 10)** | **c.2692C>T, p.Arg898Trp, rs587777659**  **(Ref. 1,4,5)** | **c.172dupA, I58Nfs*45**  **(Ref. 9)** | **c.1094T>C, p.Leu365Pro, rs587777663**  **(Ref. 2,3)** | **c.799C>T, p.Arg267*, rs369721476**  **(Ref. 2, 5, 6)** | **c.1985C>A, p.Pro662Hist** |
| Pathogenicity Prediction/Score |  |  |  |  |  |  |  |  |
| DANN | 0.9973 | Not Available | 0.9975 | 0.9992 | Not Available | 0.9987 | 0.9979 | 0.9958 |
| Mutation Taster | Disease causing | Disease causing | Disease causing | Disease causing | Disease causing | Disease causing | Disease causing automatic, Disease causing | Disease causing |
| Mutation Assessor | Low |  | Damaging | Neutral |  | Low |  | Medium |
| FATHMM | Tolerated |  | Neutral | Tolerated |  | Tolerated | Damaging | Tolerated |
| FATHMM-MKL | Damaging |  | Deleterious | Damaging |  | Damaging |  | Damaging |
| FATHMM-XF | Neutral |  | Neutral | Neutral |  | Damaging | Neutral | Neutral |
| LRT | Deleterious |  | Deleterious | Tolerated |  | Neutral | Deleterious | Deleterious |
| DEOGEN2 | Tolerated |  |  | Damaging |  | Tolerated |  | Tolerated |
| ALoFT |  |  | Recessive |  |  |  | Recessive |  |
| EIGEN | Pathogenic |  | Pathogenic | Pathogenic |  | Pathogenic | Pathogenic | Pathogenic |
| EIGEN-PC | Benign |  | Pathogenic | Benign |  | Pathogenic | Pathogenic | Pathogenic |
| SIFT | Damaging |  |  | Damaging |  | Damaging |  | Damaging |
| SIFT4G | Damaging |  |  | Damaging |  | Damaging |  | Damaging |
| PROVEAN | Neutral |  |  | Neutral |  | Damaging |  | Damaging |
| MVP | Uncertain |  |  | Uncertain |  | Uncertain |  | Uncertain |
| REVEL | Uncertain |  |  | Pathogenic |  | Pathogenic |  | Uncertain |
| PrimateAl | Tolerated |  | Tolerated | Tolerated |  | Tolerated | Tolerated | Tolerated |
| MetaSVM | Tolerated |  |  | Tolerated |  | Tolerated |  | Tolerated |
| metaLR | Tolerated |  |  | Tolerated |  | Tolerated |  | Tolerated |
|  |  |  |  |  |  |  |  |  |
| Conservation Score |  |  |  |  |  |  |  |  |
| GERP | 4.51 | 4.39 | 5.56 | 5.07 | 4.25 | 5.04 | 4.53 | 5.8 |
| PhyloP17way | 0.674 |  | 0.587 | -0.186 |  | 0.6629 | 0.597 | 0.587 |
| PhyloP30way | 1.176 |  | 1.016 | -0.3039 |  | 1.138 | 1.026 | 1.016 |
| PhyloP100way | 1.396 |  | 2.99 | 0.316 |  | 3.842 | 1.481 | 2.838 |
| PhastCons17way | 0.97 |  | 0.9879 | 0.9369 |  | 0.9969 | 0.9969 | 0.995 |
| PhastCons30way | 0.979 |  | 0.9959 | 0.537 |  | 1 | 0.999 | 1 |
| PhastCons100way | 0.9219 |  | 1 | 0.2759 |  | 1 | 1 | 1 |
| fitCons-gm (GM12878) | 0.7231 |  | 0.7025 | 0.7025 |  | 0.7231 | 0.6979 | 0.7025 |
| fitCons H1 (H1-hESC) | 0.7097 |  | 0.7437 | 0.6514 |  | 0.7097 | 0.7097 | 0.7437 |
| fitCons HU (HUVEC) | 0.7144 |  | 0.6356 | 0.6356 |  | 0.6356 | 0.6356 | 0.6356 |
| Integrated fitCons | 0.7194 |  | 0.7324 | 0.6718 |  | 0.7194 | 0.7063 | 0.7324 |
| MPC | 1.687 |  |  | 1.542 |  | 1.907 |  | 1.625 |
| bstatistic | 385 |  | 374 | 371 |  | 382 | 385 | 374 |

| Allelic Variants | | | | | | | | |
| --- | --- | --- | --- | --- | --- | --- | --- | --- |
|  | **c.1090C>T, p.Arg364***  **(Ref. 4,7)** | **c.391-392dupTT, p.Leu131Phefs*7** | **c.1094T>C, p.Leu365Pro, rs587777663**  **(Ref. 2)** | **c.2696dupG, p.Leu900Serfs*12** | **c.766delT, p.Leu256***  **(Ref. 2)** | **c.809delC, Arg271Glyfs*7** | **c.1325delA; p.Glu442Glyfs*19** | **c.1095_1102delGCGGGATGinsC, p.Arg366Leufs*7** |
| Pathogenicity Prediction/Score |  |  |  |  |  |  |  |  |
| DANN | 0.9982 |  | 0.9987 |  |  |  |  |  |
| Mutation Taster | Disease causing | Disease causing | Disease causing | Disease causing | Disease causing | Disease causing | Disease causing | Disease causing |
| Mutation Assessor |  |  | Low |  |  |  |  |  |
| FATHMM |  |  | Tolerated |  |  |  |  |  |
| FATHMM-MKL | Damaging |  | Damaging |  |  |  |  |  |
| FATHMM-XF | Neutral |  | Damaging |  |  |  |  |  |
| LRT | Deleterious |  | Neutral |  |  |  |  |  |
| DEOGEN2 |  |  | Tolerated |  |  |  |  |  |
| ALoFT | Recessive |  |  |  |  |  |  |  |
| EIGEN | Pathogenic |  | Pathogenic |  |  |  |  |  |
| EIGEN-PC | Pathogenic |  | Pathogenic |  |  |  |  |  |
| SIFT |  |  | Damaging |  |  |  |  |  |
| SIFT4G |  |  | Damaging |  |  |  |  |  |
| PROVEAN |  |  | Damaging |  |  |  |  |  |
| MVP |  |  | Uncertain |  |  |  |  |  |
| REVEL |  |  | Pathogenic |  |  |  |  |  |
| PrimateAl | Tolerated |  | Tolerated |  |  |  |  |  |
| MetaSVM |  |  | Tolerated |  |  |  |  |  |
| metaLR |  |  | Tolerated |  |  |  |  |  |
|  |  |  |  |  |  |  |  |  |
| Conservation Score |  |  |  |  |  |  |  |  |
| GERP | 5.04 | 4.625 | 5.04 | 5.07 | 4.53 | 4.53 | 4.84 | 5.006 |
| PhyloP17way | 0.597 |  | 0.6629 |  |  |  |  |  |
| PhyloP30way | 1.026 |  | 1.138 |  |  |  |  |  |
| PhyloP100way | 1.613 |  | 3.842 |  |  |  |  |  |
| PhastCons17way | 0.995 |  | 0.9969 |  |  |  |  |  |
| PhastCons30way | 0.999 |  | 1 |  |  |  |  |  |
| PhastCons100way | 0.998 |  | 1 |  |  |  |  |  |
| fitCons-gm (GM12878) | 0.7231 |  | 0.7231 |  |  |  |  |  |
| fitCons H1 (H1-hESC) | 0.6589 |  | 0.7097 |  |  |  |  |  |
| fitCons HU (HUVEC) | 0.6356 |  | 0.6356 |  |  |  |  |  |
| Integrated fitCons | 0.7194 |  | 0.7194 |  |  |  |  |  |
| MPC |  |  | 1.907 |  |  |  |  |  |
| bstatistic | 382 |  | 382 |  |  |  |  |  |

**Annex table 5.** *In silico* analysis of ARMC5 germline allelic variant from the patient (35*) with bilateral adrenal incidentaloma.

|  | c.1084C>T, p.Arg362Trp rs1385397608 |
| --- | --- |
| Pathogenicity Prediction/Score |  |
| DANN | 0.9992 |
| Mutation Taster | Disease causing |
| Mutation Assessor | Medium |
| FATHMM | Tolerated |
| FATHMM-MKL | Damaging |
| FATHMM-XF | Neutral |
| LRT | Deleterious |
| DEOGEN2 | Tolerated |
| ALoFT |  |
| EIGEN | Pathogenic |
| EIGEN-PC | Pathogenic |
| SIFT | Damaging |
| SIFT4G | Damaging |
| PROVEAN | Damaging |
| MVP | Uncertain |
| REVEL | Pathogenic |
| PrimateAl | Tolerated |
| MetaSVM | Tolerated |
| metaLR | Tolerated |
|  |  |
| Conservation Score |  |
| GERP | 5.0399 |
| PhyloP17way | 0.597 |
| PhyloP30way | 1.026 |
| PhyloP100way | 2.163 |
| PhastCons17way | 0.998 |
| PhastCons30way | 1 |
| PhastCons100way | 1 |
| fitCons-gm (GM12878) | 0.7231 |
| fitCons H1 (H1-hESC) | 0.6589 |
| fitCons HU (HUVEC) | 0.6356 |
| Integrated fitCons | 0.7194 |
| MPC | 1.5383 |
| bstatistic | 382 |

**References**

1. Guillaume Assié, M.D., Ph.D., Rossella Libé, M.D., Stéphanie Espiard, M.D., Marthe Rizk-Rabin, Ph.D., Anne Guimier, M.D., Windy Luscap, M.Sc., Olivia Barreau, M.D., Lucile Lefèvre, M.Sc., Mathilde Sibony, M.D., Laurence Guignat, M.D., Stéphanie Rodriguez, M.Sc., Karine Perlemoine, B.S., et al. ARMC5 Mutations in Macronodular Adrenal Hyperplasia with Cushing's Syndrome. N Engl J Med 2013; 369:2105-2114. DOI: 10.1056/NEJMoa1304603
2. [Guilherme Asmar Alencar](javascript:;), [Antonio Marcondes Lerario](javascript:;), [Mirian Yumie Nishi](javascript:;),[Beatriz Marinho de Paula Mariani](javascript:;), [Madson Queiroz Almeida](javascript:;), [Johanne Tremblay](javascript:;),[Pavel Hamet](javascript:;), [Isabelle Bourdeau](javascript:;), [Maria Claudia Nogueira Zerbini](javascript:;),[Maria Adelaide Albergaria Pereira](javascript:;), [Gilberto Carlos Gomes](javascript:;), [Manoel de Souza Rocha](javascript:;),[Jose Luis Chambo](javascript:;), [André Lacroix](javascript:;), [Berenice Bilharinho Mendonca](javascript:;),[Maria Candida Barisson Villares Fragoso](javascript:;). ARMC5 Mutations Are a Frequent Cause of Primary Macronodular Adrenal Hyperplasia. The Journal of Clinical Endocrinology & Metabolism, Volume 99, Issue 8, 1 August 2014, Pages E1501–E1509, <https://doi.org/10.1210/jc.2013-4237>.
3. Ulf Elbelt,corresponding author* Alessia Trovato, Michael Kloth, Enno Gentz, Reinhard Finke, Joachim Spranger, David Galas, Susanne Weber, Cristina Wolf, Katharina König, Wiebke Arlt, Reinhard Büttner, Patrick May,* Bruno Allolio,* and Jochen G. Schneider. Molecular and Clinical Evidence for an ARMC5 Tumor Syndrome: Concurrent Inactivating Germline and Somatic Mutations Are Associated With Both Primary Macronodular Adrenal Hyperplasia and Meningioma. J Clin Endocrinol Metab. 2015 Jan; 100(1): E119–E128. Published online 2014 Oct 3. doi: 10.1210/jc.2014-2648.
4. Faucz FR, Zilbermint M, Lodish MB, Szarek E, Trivellin G, Sinaii N, et al. Macronodular adrenal hyperplasia due to mutations in an armadillo repeat containing 5 (ARMC5) gene: a clinical and genetic investigation. J Clin Endocrinol Metab. 2014;99(6):E1113-9.
5. Stéphanie Espiard,* Ludivine Drougat,* Rossella Libé,* Guillaume Assié,* Karine Perlemoine, Laurence Guignat, Gaelle Barrande, Françoise Brucker-Davis, Françoise Doullay, Stephanie Lopez, Emmanuel Sonnet, Florence Torremocha, Denis Pinsard, Nathalie Chabbert-Buffet, Marie-Laure Raffin-Sanson, Lionel Groussin, Françoise Borson-Chazot, Joël Coste, Xavier Bertagna, Constantine A. Stratakis, Felix Beuschlein, Bruno Ragazzon, and Jérôme Bertherat. ARMC5 Mutations in a Large Cohort of Primary Macronodular Adrenal Hyperplasia: Clinical and Functional Consequences. J Clin Endocrinol Metab. 2015 Jun; 100(6): E926–E935. Published online 2015 Apr 8. doi: 10.1210/jc.2014-4204
6. Gagliardi L, Schreiber AW, Hahn CN, Feng J, Cranston T, Boon H, et al. ARMC5 mutations are common in familial bilateral macronodular adrenal hyperplasia. J Clin Endocrinol Metab. 2014;99(9):E1784-92.
7. Albiger, N. M, Regazzo D, Rubin B, Ferrara A. M, Rizzati S, Taschin E, Ceccato F, Arnaldi G, Pecori , Stigliano A, Cerquetti L, Grimaldi F, De Menis E, Boscaro M, Iacobone M, Occhi G, Scaroni C. A multicenter experience on the prevalence of ARMC5 mutations in patients with primary bilateral macronodular adrenal hyperplasia: from genetic characterization to clinical phenotype. Endocrine (2017) 55:959–968 DOI 10.1007/s12020-016-0956-z
8. Stéphanie Espiard. Hyperplasie macronodulaire bilatérale des surrénales. Thèse de doctorat de biologie cellulaire et moléculaire. 2016.
9. [Rego T](https://www.ncbi.nlm.nih.gov/pubmed/?term=Rego%20T%5BAuthor%5D&cauthor=true&cauthor_uid=28458897), [Fonseca F](https://www.ncbi.nlm.nih.gov/pubmed/?term=Fonseca%20F%5BAuthor%5D&cauthor=true&cauthor_uid=28458897), [Espiard S](https://www.ncbi.nlm.nih.gov/pubmed/?term=Espiard%20S%5BAuthor%5D&cauthor=true&cauthor_uid=28458897), [Perlemoine K](https://www.ncbi.nlm.nih.gov/pubmed/?term=Perlemoine%20K%5BAuthor%5D&cauthor=true&cauthor_uid=28458897), [Bertherat J](https://www.ncbi.nlm.nih.gov/pubmed/?term=Bertherat%20J%5BAuthor%5D&cauthor=true&cauthor_uid=28458897), [Agapito A](https://www.ncbi.nlm.nih.gov/pubmed/?term=Agapito%20A%5BAuthor%5D&cauthor=true&cauthor_uid=28458897).[Endocrinol Diabetes Metab Case Rep.](https://www.ncbi.nlm.nih.gov/pubmed/28458897)  *ARMC5* mutation in a Portuguese family with primary bilateral macronodular adrenal hyperplasia(PBMAH). 2017 Mar 27;2017. pii: 16-0135. doi: 10.1530/EDM-16-0135. eCollection 2017.
10. Chika Kyo,1 Takeshi Usui,2,3 Rieko Kosugi,1,2 Mizuki Torii,1 Takako Yonemoto,1,2 Tatsuo Ogawa,1 Masato Kotani,1 Naohisa Tamura,1,3 Yutaro Yamamoto,4 Takuyuki Katabami,4 Isao Kurihara,5 Kohei Saito,1,2,3,5 Naotetsu Kanamoto,6 Hidenori Fukuoka,7 Norio Wada,8 Hiroyuki Murabe,9 and Tatsuhide Inoue. ARMC5 Alterations in Primary Macronodular Adrenal Hyperplasia (PMAH) and the Clinical State of Variant Carriers. ISSN 2472-1972 October 2019 | Vol. 3, Iss. 10 doi: 10.1210/js.2019-00210 | Journal of the Endocrine Society | 1837–1846.
